# Effect of a Parent-Focused eHealth Intervention on Children’s Fruit, Vegetable, and Discretionary Food Intake (Food4toddlers): Randomized Controlled Trial

**DOI:** 10.2196/18311

**Published:** 2021-02-16

**Authors:** Margrethe Røed, Anine C Medin, Frøydis N Vik, Elisabet R Hillesund, Wendy Van Lippevelde, Karen Campbell, Nina C Øverby

**Affiliations:** 1 Department of Nutrition and Public Health University of Agder Kristiansand Norway; 2 Department of Marketing, Innovation and Organisation Ghent University Ghent Belgium; 3 Institute for Physical Activity and Nutrition Deakin University Geelong Australia

**Keywords:** toddler, child, eHealth, intervention, randomized controlled trial, fruit, vegetable, discretionary food

## Abstract

**Background:**

In Western countries, children’s diets are often low in fruits and vegetables and high in discretionary foods. Diet in early life tends to track through childhood and youth and even into adulthood. Interventions should, therefore, be delivered in periods when habitual traits are established, as in toddlerhood when children adapt to their family’s diet.

**Objective:**

In this study, we assessed the effect of the Food4toddlers eHealth intervention, which aimed to enhance toddlers’ diets by shaping their food and eating environment.

**Methods:**

The Food4toddlers randomized controlled trial was conducted in Norway in 2017-2018. Parent-child dyads were recruited through social media. In total, 298 parents completed an online questionnaire at baseline (mean child age 10.9 months, SD 1.2). Postintervention questionnaires were completed immediately after the intervention (ie, follow-up 1; mean child age 17.8 months, SD 1.3) and 6 months after the intervention (ie, follow-up 2; mean child age 24.2 months, SD 1.9). The intervention was guided by social cognitive theory, which targets the linked relationship between the person, the behavior, and the environment. The intervention group (148/298, 49.7%) got access to the Food4toddlers website for 6 months from baseline. The website included information on diet and on how to create a healthy food and eating environment as well as activities, recipes, and collaboration opportunities. To assess intervention effects on child diet from baseline to follow-up 1 and from baseline to follow-up 2, we used generalized estimating equations and a time × group interaction term. Between-group differences in changes over time for frequency and variety of fruits and vegetables and frequency of discretionary foods were assessed.

**Results:**

At follow-up 1, a significant time × group interaction was observed for the frequency of vegetable intake (*P*=.02). The difference between groups in the change from baseline to follow-up 1 was 0.46 vegetable items per day (95% CI 0.06-0.86) in favor of the intervention group. No other significant between-group differences in dietary changes from baseline to follow-up 1 or follow-up 2 were observed. However, there is a clear time trend showing that the intake of discretionary foods increases by time from less than 1 item per week at baseline to more than 4 items per week at 2 years of age (*P*<.001), regardless of group.

**Conclusions:**

A positive intervention effect was observed for the frequency of vegetable intake at follow-up 1 but not at follow-up 2. No other between-group effects on diet were observed. eHealth interventions of longer duration, including reminders after the main content of the intervention has been delivered, may be needed to obtain long-terms effects, along with tailoring in a digital or a personal form.

**Trial Registration:**

International Standard Randomized Controlled Trial Number (ISRCTN) 92980420; https://doi.org/10.1186/ISRCTN92980420

## Introduction

What toddlers eat is crucial for their health and growth, and in several western countries, young children do not meet dietary guidelines. A specific challenge is the low intake of fruits and vegetables and the high intake of sugar-sweetened beverages and snacks [1-4]. An unhealthy diet early in life increases the risk for overweight, noncommunicable diseases, and certain cancers [5,6].

The World Health Organization’s (WHO) report on ending childhood obesity [5] recommends that appropriate and context-specific nutrition information should be easily available for specific target groups and be delivered in ways that are perceived as meaningful for the users. WHO argues that such information is specifically relevant for parents of infants and toddlers. Diet in early life tends to track through childhood and youth and even into adulthood [5,7]. Interventions should, therefore, be delivered when healthy habitual traits are established in the early years, and one of these periods involves the transition from specific baby foods to eating family meals [7-10].

Parents are the primary gatekeepers of child diet in this period [11,12]. To date, few studies assessing the effect of dietary interventions targeting young children through their parents have been undertaken [13,14]. The internet is a popular source for health information among parents, and parents have reported a need for trustworthy, evidence-based, and highly accessible information sources [15-18]. Theory- and evidence-based eHealth interventions, where intervention messages are delivered to the target audience via electronic means and are easily available and accessible for the parents, may fill this information gap. eHealth interventions have the potential to reach many, can easily be changed and adapted to new groups, are available 24/7, and are cost-effective [19-21].

Parental-focused interventions with an emphasis on creating a healthy food and eating environment for the child are recommended and have shown promising results [22,23]. A healthy food environment is characterized by the accessibility and availability of healthy foods for the child and restricted access to unhealthy alternatives [22,24]. In order to create a healthy eating environment, it is essential to incorporate health-promoting feeding practices, such as healthy modeling and repeated exposure to healthy foods [7].

The aim of this study was to examine the effect of a parent-focused eHealth intervention on the child’s diet assessed at two time points postintervention. We hypothesized that, compared with the control group, the children in the intervention group would develop a more frequent and varied intake of fruits and vegetables and less frequent intake of discretionary foods from baseline to postintervention.

## Methods

### Design and Study Population

This study used data from the Food4toddlers randomized controlled trial (RCT), an eHealth intervention aiming to promote a healthy food and eating environment for toddlers. Details of the intervention’s design and components have been previously published [25]. The study was a 2-armed RCT involving 298 parent-child dyads. This eHealth intervention was conducted in Norway in 2017-2018. Data were collected at baseline, after 6 months (ie, follow-up 1: postintervention), and after 12 months (ie, follow-up 2: 6 months postintervention). Parents in the intervention group were provided with access to the Food4toddlers website for a period of 6 months after completing the baseline questionnaires. Log-in instructions for the website were sent by email, and up to three reminders were sent to nonresponders. Informed consent from the parents was obtained when they signed in online for participation in the study. A completed CONSORT-EHEALTH (Consolidated Standards of Reporting Trials of Electronic and Mobile HEalth Applications and onLine TeleHealth) V1.6.1 checklist is available in Multimedia Appendix 1. Research clearance was obtained from the Norwegian Centre for Research Data on June 8, 2016 (reference No. 48643).

Between August 2017 and January 2018, 404 parents of infants and toddlers from across Norway were recruited through a tailored advertisement on Facebook and accepted to participate by signing in at the study home page [26]. Parents of children born between June 2016 and May 2017 were eligible for participation if they were literate in Norwegian. In the case of twins, the parent reported on behalf of the oldest child. All sociodemographic and behavioral data were collected at baseline and follow-up time points using the online survey software SurveyXact (Rambøll) [27]. Up to three email reminders were sent in the absence of a response. Survey items completed by parents concerning children’s food intake, parental feeding practices, and demographic data were included in the analysis.

Participants were randomized and allocated to either an intervention or a control group after the baseline data collection. A randomization list was generated in SPSS Statistics for Windows, version 25.0 (IBM Corp), by one of the researchers (NØ) and implemented by the first author (MR). The follow-up questionnaires replicated the baseline questionnaires but also included questions on intervention website use; only the intervention group completed these questions at follow-up 1.

### Intervention Development

This Food4toddlers study was developed using the basic steps from the Model of Planned Promotion for Population Health [28]. This intervention is in line with social cognitive theory, which targets the linked relationship between the person, the behavior, and the environment [29] with an emphasis on how to promote action rather than motivation only [28]. The participants were encouraged to have core foods available, especially vegetables and fruit, both in their home and on the child’s plate. The opposite was encouraged for discretionary (ie, noncore) foods and beverages. Food4toddlers was developed in a cocreation process with health care nurses, parents of toddlers, and students and staff at the University of Agder, Norway. Key elements in this process included several individual and group interviews with stakeholders and the inclusion of students in developing and pilot-testing the website. The Food4toddlers eHealth intervention included a website with four main elements: (1) modules, including two to four lessons, covering an introduction and seven topics on promoting healthy food and eating environments for the child; (2) recipes; (3) a discussion forum; and (4) highlighted information about food and beverages, called *Good to know*. In addition, when accessing the Food4toddlers website, a video appeared with information about the study and its focus on how important just a small weekly increase in vegetable consumption may be for the child’s long-term health. Small behavioral changes were highlighted with the aim of making the messages easier for the parent to accomplish [30]. The modules had activity elements, such as a quiz or a game, and visual elements that supported the information. During the intervention period, the participants received weekly emails, each containing a link to a new lesson (20 times), thus expanding the content of the intervention. Some lessons were more comprehensive than others, but the estimated time to complete an average lesson was 10 minutes. The Food4toddlers website was available on smartphones and other tablets in the form of a mobile app, in addition to computers.

### Measures and Outcomes

#### Overview

The importance of fruit and vegetable intake for lifelong health is well-documented [3,4], and a diet rich in fruits and vegetables and limited in discretionary foods is the cornerstone of a high-quality diet [2,31-34]. We wanted to measure the frequency of these foods along with the variety of fruits and vegetables, which is less frequently measured [35] and has been shown to be an indicator of preschoolers’ overall diet quality [36]. Our previous research using baseline data from the Food4toddlers intervention revealed different patterns in fruit versus vegetable consumption [37]. Therefore, we wanted to further examine this distinction for the intervention’s effect and examine the intake of discretionary foods to elaborate on both core and noncore dietary effects. We constructed three separate scales to assess the consumption frequency of vegetables, fruits, and discretionary foods, respectively. Food variety scale scores were calculated separately for vegetables and fruits.

#### Child Food Intake

Child food intake in this study was assessed by a food frequency questionnaire (FFQ), based on questionnaires previously used in the population-based Norwegian Mother, Father and Child Cohort Study [38] and the nationwide Norwegian diet survey among 12-month-old children [39]. These questionnaires have been previously validated in toddlers [40,41]. Using both questionnaires, we were able to cover a more extensive selection of foods, but different scales made the comparison more difficult. Of the 59 FFQ items in the questionnaire, we used three food groups, comprising 33 items in total.

#### Assessment of Fruit and Vegetable Intake

Questions covering the intake of fruits and vegetables commonly consumed in Norway [42] included the following: “How often does your child eat the following fruits/vegetables nowadays?” The food items presented included fresh, cooked, or squeezed fruits and vegetables and both homemade and commercially produced variants. In total, 13 vegetables (ie, *carrot, rutabaga, sweet potato, cauliflower, broccoli, green salad, spinach, cucumber, tomato, corn, sweet pepper, pea,* and *other*) and 11 fruits (ie, *orange, banana, apple, pear, plum, grapes, kiwi, melon, mango, berries,* and *other*) were listed.

A 6-point scale, ranging from *never* to *several times a day*, was used with the following response options and recoded into times per week: never or less than once a week = 0, one to three times a week = 2, four to six times a week = 5, once a day = 7, two times a day = 14, and three times or more per day = 24.5. Similar recoding has been done by others [43-46]. We calculated a combined score of total vegetable intake and another for total fruit intake (ie, frequency per day).

The same items, as previously described for the frequency of vegetables and fruits, were used to calculate variety scores of eaten (coded 1) and not eaten (coded 0) vegetables (13 items) and fruits (11 items).

#### Assessment of Discretionary Foods and Beverages

The questions on the consumption frequency of discretionary foods included the following: “How often does your child eat the following foods nowadays?” The following food groups were assessed: (1) cakes, waffles, and sweet biscuits; (2) desserts and ice cream; (3) chocolate; (4) candy and such; and (5) chips. A 6-point scale was used, ranging from *never* to *several times a day*. The response options were recoded into times per week: never = 0, less than once a week = 0.5, one to three times a week = 2, four to six times a week = 5, one to two times a day = 10.5, and three times or more per day = 24.5.

Beverage intake was assessed with the following question: “How often does your child drink the following drinks nowadays?” Two sugar-sweetened beverages were included. The response options were recoded into daily intake: never/seldom = 0, one to three times a week = 0.29, four to six times a week = 0.71, one per day = 1, two per day = 2, three per day = 3, four per day = 4, and five or more per day = 6. They were then coded into times per week (ie, multiplied by 7) to be consistent with the snack score. Subsequently, we calculated the sum of the combined frequency of intake of discretionary foods per week, including five snack items and two beverage items.

#### Assessment of Demographics and Use of the Website

Parents reported the following at baseline: child’s gender, child’s date of birth, whether they lived together with the child’s other parent, and their own height, weight, date of birth, and education level. The parent’s BMI was calculated from self-reported height and weight (kg/m^2^). The categories for parental education level were as follows: primary school or less; primary school plus one year of, for example, folk high school; high school; vocational school; upper secondary school or less; college or university (≤4 years); college or university (>4 years); other; and don’t know. These categories are similar to categories used by others in Norway [39]. The education level was dichotomized: none or up to 4 years of higher-level education and more than 4 years of higher-level education. This cutoff was used since the groups without college or university education were very small (total 11.3%) and since we know that a healthy lifestyle increases for every year of education [47].

From the website, we registered the number of lessons (ie, 22 in total) that the participants in the intervention group had completed. The lessons comprised two to four pages and all of them had to be visited for a lesson to be registered as completed. Lesson number 7 had an element that was only available via a computer; all other lessons were available on different devices.

### Statistics

The sample size was calculated for one of the primary outcomes: child diet quality. Because no data on healthy eating scores for Norwegian toddlers are available, the calculation for this study was based on the study of Angelopoulos et al [48]. They used a healthy diet score of 10 components to assess child diet and observed a mean score of 60.5 (SD 9.2). A 3-point difference in score between the control and intervention groups was considered relevant from a public health perspective. From this, we estimated that 142 children in each group would be required to demonstrate statistical significance with a statistical power of 80% and α level of 5%. Assuming a 40% loss to follow-up, we aimed to recruit 237 parents in each group.

Means with standard deviations for continuous variables and frequencies and percentages for categorical variables were reported for baseline characteristics.

We used generalized estimating equations (GEEs) to determine whether the intervention had an effect on child diet from baseline to follow-up 1 and from baseline to follow-up 2. GEEs are suited for identifying how much a sample’s average response changes with a one-unit increase in a covariant, which means that all respondents can be included in the analyses even though there are missing responses on the follow-up questionnaires [49]. This method also takes into account the problem with individual correlated data [49]. Frequency of intake (ie, vegetables, fruits, and discretionary foods) and variety of intake (ie, vegetables and fruits) were included as dependent variables in separate models. An interaction term between group (ie, intervention vs control) and time (ie, baseline vs postintervention) was entered into all models to examine the possible effects of the intervention. Specifically, we investigated whether changes in dietary intake from baseline to postintervention periods (ie, follow-up 1 and follow-up 2) differed significantly between the control and intervention groups. An unstructured covariance matrix and robust estimates of the standard error were used. All models were adjusted for child gender and age as well as for parental BMI, education level, and age reported at baseline. We selected covariates based on previous research on determinants for vegetable and fruit intake [50] and in line with the protocol for the study [25]. We ran *t* tests and Mann-Whitney *U* tests as sensitivity analyses, using complete cases and the difference between baseline and follow-up 1 values and baseline and follow-up 2 values for all outcome variables. The intention‐to‐treat principle was used in the analyses [51]. All analyses were conducted in SPSS Statistics for Windows, version 25.0 (IBM Corp), except for GEEs, which were run in Stata, version 16 (StataCorp LLC). Statistical significance level was set at *P*≤.05.

### Availability of Data and Materials

The data set supporting the conclusions of this article will be available in the University of Agder Open Research repository [52].

### Ethics Approval, Trial Registration, and Consent to Participate

This trial was approved by the Norwegian Centre for Research Data on June 29, 2017 (reference No. 48643). This trial was registered at the International Standard Randomised Controlled Trial Number (ISRCTN) registry on September 13, 2017 (trial No. 92980420). Written consent was obtained from all parents on the study home page [26] when they chose to sign up for participation.

## Results

### Characteristics of the Study Sample

**Figure 1:**
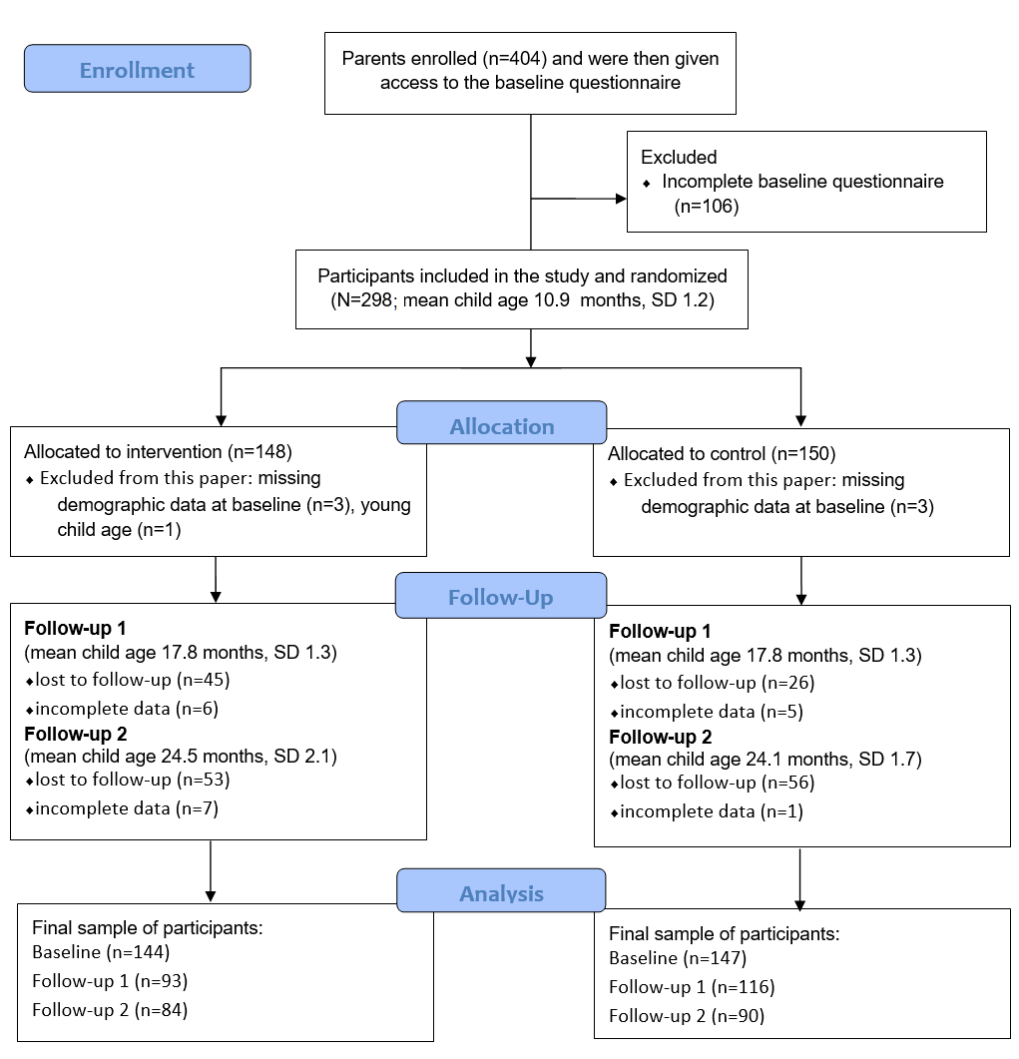
CONSORT (Consolidated Standards of Reporting Trials) diagram for the Food4toddlers randomized controlled trial study.

Figure 1 shows the flow of participants in the study. Of the 404 parents that signed up to participate, 298 (73.8%) completed the baseline questionnaire and were included in the study. After the baseline data collection, parents were randomized into the intervention group (148/298, 49.7%) or the control group (150/298, 50.3%). In total, 1 child was erroneously included in the study because he or she was too young and 6 participants had missing data on demographic variables at baseline (ie, parental age, BMI, or education level); they were excluded from the analyses in this paper. Table 1 shows group comparisons of baseline characteristics and food intake measures between participants in the intervention and control groups. There were no significant differences between the groups. Tables 2 and 3 show group comparisons of baseline characteristics between participants retained in this study at follow-up 1 (ie, immediately after the intervention) and follow-up 2 (ie, 6 months postintervention), respectively, and those who were lost to follow-up or had missing data on outcome variables at these time points.

**Table 1:** Baseline characteristics of parents and children in the intervention and control groups and children’s food intake measures.

Characteristics and food intake measures			Total (n=291)	Control (n=147)	Intervention (n=144)
**Demographic characteristics**					
	**Parents**				
		Mother^a^, n (%)	287 (98.6)	147 (100)	140 (97.2)
		Father, n (%)	4 (1.4)	0 (0)	4 (2.8)
		Age (years), mean (SD)	31.7 (4.2)	31.8 (3.9)	31.5 (4.4)
		Height (cm), mean (SD)	168.4 (5.9)	168.1 (5.9)	168.7 (6.0)
		Weight (kg), mean (SD)	70.7 (14.2)	71.0 (14.8)	70.4 (13.7)
		BMI (kg/m^2^), mean (SD)	24.9 (4.6)	25.1 (4.8)	24.7 (4.4)
		Two-parent household, n (%)^b^	288 (99.0)	144 (98.0)	144 (100)
		Total number of household members, mean (SD)	3.6 (0.9)	3.7 (0.9)	3.6 (1.0)
		Born in Norway, n (%)	250 (86.2)^c^	122 (83.0)^c^	128 (88.9)
		Education: ≤4 years college or university or lower, n (%)	132 (45.4)	70 (47.6)	62 (43.1)
	**Children**				
		Age (months), mean (SD)	10.9 (1.2)	10.8 (1.2)	10.9 (1.2)
		Child’s sex: female, n (%)	129 (44.3)	63 (42.9)	66 (45.8)
**Children’s food intake at baseline, mean (SD)**					
	**Vegetables (13 items)**				
		Frequency (times/day)	3.2 (1.6)	3.1 (1.5)	3.2 (1.7)
		Variation (number/week)	7.2 (2.6)	7.2 (2.5)	7.2 (2.7)
	**Fruits (11 items)**				
		Frequency (times/day)	2.8 (1.6)	2.7 (1.4)	2.9 (1.8)
		Variation (number/week)	5.8 (2.2)	5.7 (2.2)	5.9 (2.2)
	**Discretionary foods (7 items)^d^**				
		Frequency (times/week)	0.8 (1.4)	0.8 (1.4)	0.8 (1.4)

^a^Included co-mothers and foster mothers.

^b^Lived together with the other parent.

^c^One missing case.

^d^Included five unhealthy snack items and two sugar-sweetened beverages.

**Table 2:** Differences in baseline characteristics and food intake between participants who remained in the study and those lost to follow-up 1.

Demographic characteristics and food intake measures			At follow-up 1 (all participants) (n=291)					Lost to follow-up 1 from intervention and control groups^a^ (n=82)							
			Retained in study (n=209)		Lost to follow-up 1^a^ (n=82)		*P* value^b^		Control (n=31)		Intervention (n=51)		*P* value^b^		
**Demographic characteristics**															
	**Parents**														
		Mother^c^, n (%)	206 (98.6)		81 (99)		N/A^d^		31 (100)		50 (98)		N/A		
		Father, n (%)	3 (1.4)		1 (1)		N/A		0 (0)		1 (2)		N/A		
		Age (years), mean (SD)	31.8 (4.1)		31.5 (4.4)		.66		32.6 (4.2)		30.9 (4.4)		.09		
		Height (cm), mean (SD)	168 (5.8)		168 (6.0)		.74		168 (5.9)		169 (6.1)		.48		
		Weight (kg), mean (SD)	71.0 (15.0)		69.8 (12.0)		.52		67.6 (11.6)		71.2 (12.2)		.19		
		BMI (kg/m^2^), mean (SD)	24.9 (4.9)		24.6 (3.8)		.55		24.0 (3.6)		25.0 (3.9)		.24		
		Two-parent household, n (%)^e^	207 (99.0)		81 (99)		.82		30 (99)		51 (100)		.20		
		Total number of household members, mean (SD)	3.6 (1.0)		3.7 (0.8)		.37		3.9 (0.8)		3.6 (0.8)		.08		
		Born in Norway, n (%)	177 (84.7)		73 (90)^f^		.23		26 (87)^f^		47 (92)		.42		
		Education: ≤4 years of college or university or lower, n (%)	93 (44.5)		39 (48)		.64		15 (48)		24 (47)		.91		
	**Children**														
		Age (months), mean (SD)	10.8 (1.2)		11.0 (1.4)		.13		10.8 (1.7)		11.2 (1.2)		.24		
		Child’s sex: female, n (%)	90 (43.1)		39 (48)		.49		12 (39)		27 (53)		.21		
**Children’s food intake at baseline, mean (SD)**															
	**Vegetables (13 items)**														
		Frequency (times/day)	3.2 (1.6)		3.0 (1.6)		.36		2.9 (1.5)		3.1 (1.7)		.28		
		Variation (number/week)	7.4 (2.5)		6.9 (2.7)		.20		6.7 (2.6)		7.0 (2.8)		.64		
	**Fruits (11 items)**														
		Frequency (times/day)	2.9 (1.6)		2.7 (1.6)		.43		2.9 (1.7)		2.6 (1.7)		.48		
		Variation (number/week)	5.9 (2.2)		5.6 (2.2)		.39		5.6 (2.0)		5.7 (2.4)		.66		
	**Discretionary foods (7 items)^g^**														
		Frequency (times/day)	0.8 (1.4)		0.9 (1.4)		.48		0.8 (1.2)		1.0 (1.5)		.78		

^a^Participants who were lost to follow-up or had incomplete outcome data at follow-up 1.

^b^Calculated by the Pearson chi-square test or *t* test.

^c^Included co-mothers and foster mothers.

^d^N/A: not applicable; it was not relevant to the study to calculate *P* values for gender items.

^e^Lived together with the other parent.

^f^One missing case.

^g^Included five unhealthy snack items and two sugar-sweetened beverages.

**Table 3:** Differences in baseline characteristics and food intake between participants who remained in the study and those lost to follow-up 2.

Demographic characteristics and food intake measures			At follow-up 2 (all participants) (n=291)			Lost to follow-up 2 from intervention and control groups^a^ (n=117)		
			Retained in study (n=174)	Lost to follow-up 2^a^ (n=117)	*P* value^b^	Control (n=57)	Intervention (n=60)	*P* value^b^
**Demographic characteristics**								
	**Parents**							
		Mother^c^, n (%)	171 (98.3)	116 (99.1)	N/A^d^	57 (100)	59 (98)	N/A
		Father, n (%)	3 (1.7)	1 (0.9)	N/A	0 (0)	1 (2)	N/A
		Age (years), mean (SD)	32.0 (4.0)	31.2 (4.4)	.10	32.0 (4.1)	30.5 (4.6)	.07
		Height (cm), mean (SD)	169 (5.8)	168 (6.0)	.05	168 (6.2)	168 (5.8)	.89
		Weight (kg), mean (SD)	71.3 (14.7)	69.8 (13.4)	.37	69.1 (13.6)	70.6 (13.4)	.53
		BMI (kg/m^2^), mean (SD)	25.0 (4.9)	24.8 (4.2)	.76	24.4 (3.9)	25.1 (4.5)	.37
		Two-parent household, n (%)^e^	173 (99.4)	115 (98.3)	.35	55 (96)	60 (100)	.14
		Total number of household members, mean (SD)	3.6 (1.0)	3.7 (0.9)	.66	3.8 (1.0)	3.6 (0.7)	.17
		Born in Norway, n (%)	154 (88.5)	96 (82.1)	.16	42 (75)	54 (90)	.03
		Education: ≤4 years of college or university or lower, n (%)	67 (38.5)	65 (55.6)	.004	30 (53)	35 (58)	.54
	**Children**							
		Age (months), mean (SD)	10.8 (1.2)	11.0 (1.2)	.14	11.0 (1.4)	11.1 (1.1)	.60
		Child’s sex: female, n (%)	73 (42.0)	56 (47.9)	.32	27 (47)	29 (48)	.92
**Children’s food intake at baseline, mean (SD)**								
	**Vegetables (13 items)**							
		Frequency (times/day)	3.2 (1.6)	3.0 (1.5)	.27	3.2 (1.4)	2.9 (1.6)	.35
		Variation (number/week)	7.3 (2.6)	7.1 (2.7)	.47	7.3 (2.4)	6.9 (2.9)	.35
	**Fruits (11 items)**							
		Frequency (times/day)	2.9 (1.6)	2.8 (1.7)	.65	2.9 (1.4)	2.6 (1.9)	.36
		Variation (number/week)	5.9 (2.2)	5.7 (2.3)	.47	5.8 (2.1)	5.6 (2.4)	.54
	**Discretionary foods (7 items)^f^**							
		Frequency (times/day)	0.7 (1.3)	0.9 (1.5)	.18	0.9 (1.4)	1.0 (1.6)	.87

^a^Participants who were lost to follow-up or had incomplete outcome data at follow-up 2.

^b^Calculated by the Pearson chi-square test or *t* test.

^c^Included co-mothers and foster mothers.

^d^N/A: not applicable; it was not relevant to the study to calculate *P* values for gender items.

^e^Lived together with the other parent.

^f^Included five unhealthy snack items and two sugar-sweetened beverages.

At the follow-up 1 time point, 71 participants were lost to follow-up and 11 had incomplete data on outcome variables. At the follow-up 2 time point, 109 were lost to follow-up and 8 had incomplete outcome data. The number of participants that completed the baseline questionnaire was 298, and 291 (97.7%) were included in our analyses. Of these, 209 (71.8%) completed the follow-up 1 questionnaire and 174 (59.8%) completed the follow-up 2 questionnaire.

Mean parental age at baseline was 31.7 years (SD 4.2) (see Table 1). Most participants were mothers (287/291, 98.6%), lived in two-parent households (288/291, 99.0%), and were born in Norway (250/290, 86.2%). Other characteristics that are not listed in the table are as follows: the mean age of the child at follow-up 1 was 17.8 months (SD 1.23; n=209) and at follow-up 2 was 24.2 months (SD 1.68; n=174). All 19 Norwegian counties were represented in the study sample. We observed a higher proportion of participants from the south of Norway compared to the national population data [53].

The infants had a frequency daily intake of 3.2 (SD 1.6) items of vegetables and 2.8 (SD 1.6) items of fruit. For discretionary food, the weekly intake was less than 1 item (mean 0.8, SD 1.4) at baseline. The participating children ate a more varied range of vegetables (mean 7.2 per week, SD 2.6) compared to fruits (mean 5.8 per week, SD 2.2).

To get an overview of the baseline characteristics of participants who remained in the study and those who were lost to follow-up, Tables 2 and 3 present the baseline characteristics of these participants at the two follow-up time points. A comparison between all participants is presented, including how many of those lost to follow-up adhered to the intervention or control group. Of the 82 participants who did not participate in the follow-up 1 time point (see Table 2), 51 (62%) were from the intervention group and 31 (38%) were from the control group.

At follow-up 2, the number of nonresponders was comparable in the two groups (ie, 57/117, 48.7%, in the control group and 60/117, 51.3%, in the intervention group). Participants with a higher education level were more likely to complete the follow-up 2 questionnaires (*P*=.004).

Table 4 shows how many participants out of 144 in the intervention group completed each of the 22 lessons on the Food4toddlers website. The first two lessons were available when the participants got access to the website. After that, a new lesson was delivered every week. Lesson 1 was an information lesson (eg, how to navigate the website and information about the study), and lesson 7 had a gaming element included that was only accessible from a computer and not on mobile devices. Few parents completed this lesson (21/144, 14.6%). The number of parents out of 144 who completed lessons ranged from 21 (14.6%) to 87 (60.4%). We saw a general drop in parents completing lessons over time.

**Table 4:** Number of participants in the intervention group who completed lessons.

Lesson No.	Number of intervention group participants who completed each lesson (n=144), n (%)
1	68 (47.2)
2	87 (60.4)
3	78 (54.2)
4	70 (48.6)
5	63 (43.8)
6	60 (41.7)
7	21 (14.6)
8	60 (41.7)
9	63 (43.8)
10	53 (36.8)
11	46 (31.9)
12	52 (36.1)
13	54 (37.5)
14	59 (41.0)
15	52 (36.1)
16	49 (34.0)
17	45 (31.3)
18	45 (31.3)
19	45 (31.3)
20	36 (25.0)
21	32 (22.2)
22	29 (20.1)

### Dietary Outcomes

At follow-up 1, a significant time × group interaction was observed for frequency of vegetable intake (*P*=.02); see adjusted measures in Table 5. The between-group difference in the change from baseline to follow-up 1 was 0.46 items per day (95% CI 0.06-0.86), showing a larger increase in the frequency of vegetable intake in the intervention group compared to the control group. No other significant differences in dietary changes from baseline to follow-up 1 or from baseline to follow-up 2 were observed between the groups.

**Table 5:** Intervention effects of the Food4toddlers study on food intake outcomes from baseline to both follow-up time points (n=291).

Intervention effects			Baseline to follow-up 1^a^		Baseline to follow-up 2^a^	
			Mean change estimate (95% CI)	*P* value	Mean change estimate (95% CI)	*P* value
**Unadjusted**						
	**Vegetables**					
		Frequency (times/day)	0.44 (0.04 to 0.84)	.03	0.30 (–0.14 to 0.74)	.18
		Variation (number/week)	0.56 (–0.08 to 1.19)	.09	0.69 (0.04 to 1.43)	.06
	**Fruits**					
		Frequency (times/day)	0.04 (–0.45 to 0.54)	.87	–0.07 (–0.62 to 0.48)	.81
		Variation (number/week)	0.07 (–0.51 to 0.66)	.80	–0.17 (–0.82 to 0.49)	.62
	**Discretionary foods**					
		Frequency (times/day)	–0.10 (–1.20 to 1.00)	.85	0.05 (–1.02 to 1.11)	.93
**Adjusted^b^**						
	**Vegetables**					
		Frequency (times/day)	0.46 (0.06 to 0.86)	.02	0.32 (–0.12 to 0.75)	.15
		Variation (number/week)	0.60 (–0.04 to 1.23)	.07	0.73 (–0.01 to 1.46)	.05
	**Fruits**					
		Frequency (times/day)	0.03 (–0.47 to 0.52)	.91	–0.10 (–0.64 to 0.44)	.71
		Variation (number/week)	0.09 (–0.50 to 0.67)	.78	–0.18 (–0.84 to 0.48)	.60
	**Discretionary foods**					
		Frequency (times/day)	–0.07 (–1.17 to 1.02)	.89	0.08 (–0.98 to 1.14)	.89

^a^Mean change in frequency or variety of vegetables, fruits, or discretionary foods from baseline to the postinterventions (follow-up 1 or 2) between the control and intervention groups.

^b^Adjusted for child age and gender and parental BMI, education level, and age at baseline.

**Figure 2:**
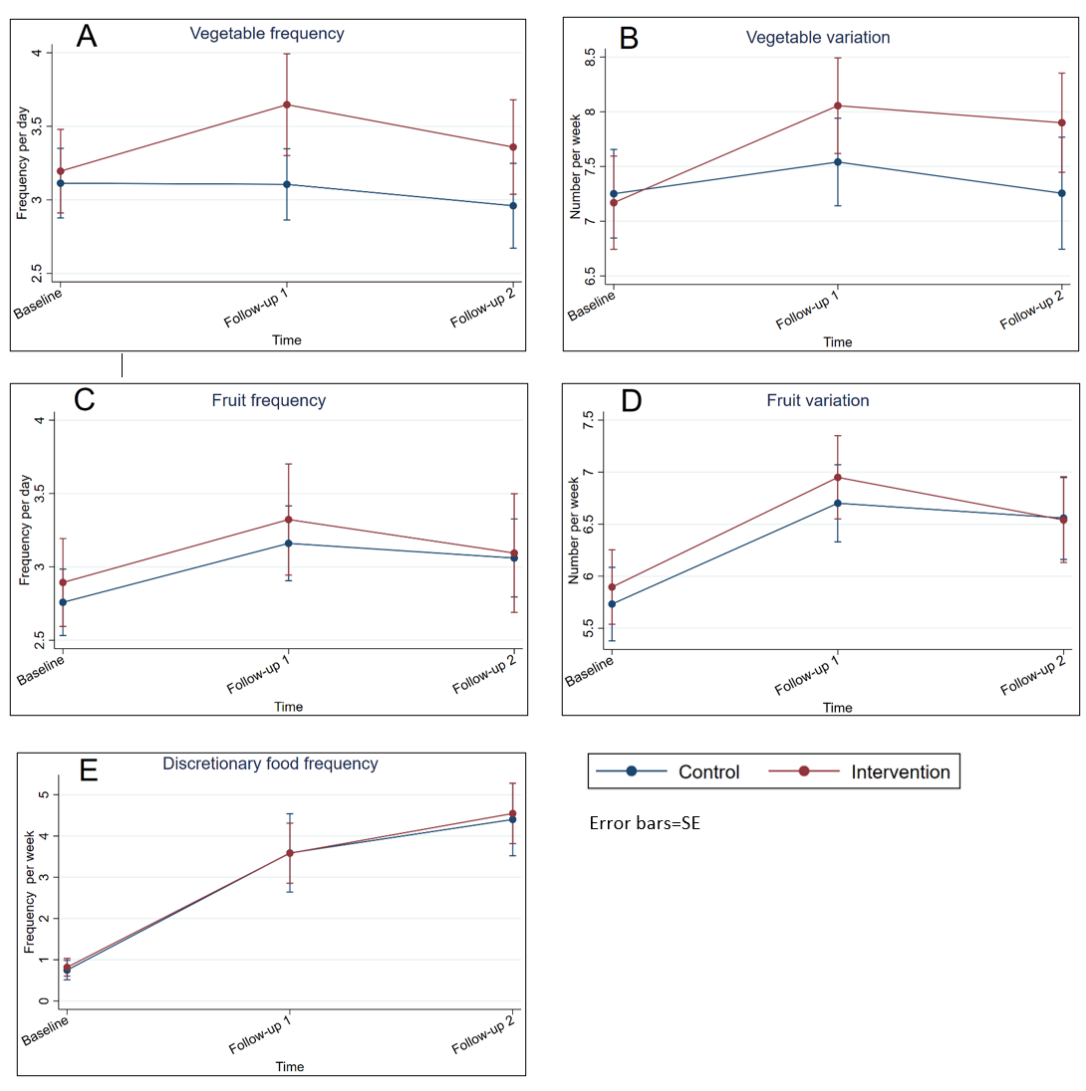
Estimated marginal means for children's food intake at baseline, follow-up 1, and follow-up 2 for the frequency of vegetables, fruits, and discretionary foods, and variety of vegetables and fruits. Values are adjusted for child age and gender and parental BMI, education level, and age reported at baseline.

The change in frequency of vegetable intake from baseline to the follow-up time points for the intervention group and the control group are presented in Figure 2A.

Estimated marginal means (EMMs) for the intervention group showed that the vegetable intake of 3.20 times per day (SE 0.15) at baseline increased to 3.65 times per day (SE 0.18) at follow-up 1. There was no change in frequency of vegetable intake in the control group from baseline (EMM 3.11 times per day, SE 0.12) to follow-up 1 (EMM 3.11 times per day, SE 0.12). A small decrease in frequency was observed from follow-up 1 to follow-up 2 in both groups: an EMM vegetable intake of 2.96 times per day (SE 0.15) was observed for the control group and 3.36 (SE 0.16) was observed for the intervention group. There was no significant time trend from baseline to the follow-up time points.

A similar trend was observed for the variety score of vegetables (see Figure 2B), although the group × time interactions were only borderline significant. Specifically, the group difference in the change from baseline to follow-up 1 was 0.60 vegetables tasted per week (*P*=.07) and from baseline to follow-up 2 was 0.73 vegetables tasted per week (*P*=.05) (see Table 5). Moreover, regarding vegetable variety, the EMM of the control group was 7.25 (SE 0.21) at baseline, 7.54 (SE 0.20) at follow-up 1, and 7.26 (SE 0.26) at follow-up 2; for the intervention group, the EMM was 7.17 (SE 0.22) at baseline, 8.06 (SE 0.22) at follow-up 1, and 7.90 (SE 0.23) at follow-up 2. No significant time trend from baseline to the follow-up time points was observed.

There were no significant between-group differences in change in the frequency nor variety of fruit intake from baseline to follow-up 1 and from baseline follow-up 2 (see Figure 2C and D). There was a significant time trend for fruit frequency from baseline to follow-up 1 (*P*=.002) and borderline significance from baseline to follow-up 2 (*P*=.052). For variety of fruit, a significant time trend was seen from baseline to both follow-up time points (*P*<.001).

No intervention effect was observed for the intake of discretionary foods, as there was no significant between-group difference in the change in intake frequency from baseline to either of the two follow-up time points. However, the intake of discretionary foods increased significantly (*P*<.001) over time (see Figure 2E). Specifically, at baseline, the intake was less than 1 item per week for both groups, which increased to 3.6 items per week in both groups (control EMM 3.6 items per week, SE 0.48; and intervention EMM 3.6 items per week, SE 0.37) at follow-up 1; this later increased to 4.4 (SE 0.45) items per week in the control group and 4.5 (SE 0.37) items per week in the intervention group at follow-up 2.

The sensitivity analyses—*t* tests and Mann-Whitney *U* tests—using complete cases showed results in line with the GEE analyses, except for the results for vegetable variation, which were no longer borderline significant.

## Discussion

### Principal Findings

In this study, we observed that giving parents access to an eHealth intervention during toddlerhood increased their children’s vegetable consumption frequency. The intervention effect was attenuated and no longer significant 6 months postintervention. A borderline significant effect for variety of vegetable intake in favor of the intervention group was observed at both time points. For fruits and discretionary foods, there were no intervention effects.

Although the intervention promoted a higher consumption of both vegetables and fruits, and lower consumption of discretionary foods, the vegetable promotion was the main focus in the Food4toddlers study, which may explain our findings. Specifically, the intervention focused on vegetables from the start; on the front page of the website and in the first lessons that the participants were able to access, vegetable promotion was central. Interest in the intervention website was highest at the start of the program, which was also seen in other web-based programs [54]. The video on the front page of the Food4toddlers website focused on how important just a small weekly increase in vegetable consumption may be for children’s long-term health. It is possible that those who accessed the website watched this video and that this may have motivated improved vegetable consumption. There may also be more room for improvement in vegetable intake relative to fruit or discretionary food intake in this age group. A British study found a larger variety of vegetables in commercially prepared dinners than in their home-cooked recipe counterparts [55]. Parents tend to serve more commercially prepared dinners at the age of 1 year than they do later [39,56]. This may explain why the variety of vegetables did not increase by age, even though children eat larger portions of foods by age [39,56]. A known reason for lack of variety is the age-specific trait of rejection of new foods—food neophobia—that peaks around 2 years of age [57]. In order to create a healthy eating environment, this study focused on the importance of repeated exposure as a means to enhance the acceptance of new foods before that age. There was a borderline significant difference between the groups for vegetable variety in our study, at both time points, that may indicate that children in the intervention group obtained a higher acceptance of these foods before the age of 2 years, which may persist over time [7]. While there are no studies directly comparable to this study, some have reported dietary outcomes of eHealth interventions targeting older or younger children. The Swedish *Mobile-based intervention intended to stop obesity in preschoolers* (MINISTOP) mobile health (mHealth) intervention [58] reported no effect on vegetable consumption. The MINISTOP study targeted parents of 4-year-olds, and the intervention group got access to an app for 6 months that focused on a healthy diet and physical activity. The results from the Australian Time2bHealthy study for vegetable consumption were in line with the MINISTOP study. Time2bHealthy delivered an 11-week web course on healthy lifestyle to the intervention group, followed by fortnightly emails for 3 months. They targeted parents of 2- to 5-year-old children with BMI values at or above the 50^th^ percentile for their age, and the participants got individual feedback from a dietitian. Our intervention targeted parents with younger children in a period where dietary habits are established, which may explain the positive results for vegetables in our study. A Norwegian eHealth RCT intervention, *Early food for future health*, delivered monthly videos on child-feeding to parents of infants (6-12 months of age) [59] and found an intervention effect for vegetable variation [59]. They made a composite score of fruit and vegetable frequency, which also showed improvement in intake [59]. A similar score was used in two studies targeting older children [60,61] showing positive results, contrary to no reported effect found in an mHealth study targeting infants [20]. A composite healthy lifestyle score was assessed in the MINISTOP study that showed a positive intervention effect [58].

Both the intervention and the control groups increased their intake of fruit over time, but no intervention effects between the groups were seen. The lack of effect on fruit intake contrary to vegetable intake may be explained by the differences between the two types of foods in terms of skills and time needed for preparation, consumption patterns, and the parents’ readiness to make changes [62-64]. Few preparations are necessary to give the child a fruit as a snack or in a smoothie, and fruits are more easily accepted by children than vegetables due to their sweet taste [65]. The children may have tasted and accepted a variety of fruits before the intervention period started, and improvements may be hard to obtain. The lack of an intervention effect on fruit consumption has been observed in comparable studies [58,59,66].

In contrast with our findings and those from other studies [59,67], the Time2bHealthy study showed an effect on discretionary foods in favor of the intervention group [66]. A review exploring both traditional and eHealth interventions aiming to reduce sugar‐sweetened beverages among young children (<5 years of age) found that success was more likely if interventions were multicomponent, targeted vulnerable populations, and had a high intervention intensity and contact time [68]. The Time2bHealthy study was conducted in line with these success factors, which may explain the positive results. The MINISTOP study found an intervention effect on sweetened beverage consumption in favor of the intervention group [67]. The offering of discretionary foods was low at baseline in the Food4toddlers study and increased at the follow-up time points in both groups. However, the intake remained relatively low when compared with other studies [69,70]. The increase in both groups over time may be explained by the fact that children tend to incorporate the rest of their family’s eating patterns, including more discretionary food, during the second year of life (eg, ice cream in the summer and biscuits as snacks).

Findings from this study and other similar studies show that digital interventions may be effective in improving some aspects of dietary intake. However, for most parent-focused eHealth studies, long-term retention of effects have not been observed [17,58]. One interesting exception is the long-term effect on discretionary foods in the Time2bHealthy study [66]. The lack of long-term effects is a challenge for eHealth interventions aimed at lifestyle behavior, in general [71], and specifically for parent-focused, traditional and online, obesity prevention interventions [13,72]. A duration of 6 months or shorter is common in parent-focused eHealth interventions [13,25,73]. A longer duration might contribute to maintained effects over time [13,71,74]. Further, including short and thematically narrow “booster sessions” after the end of more intensive intervention sessions have shown promising results [72] and may also maintain the effects of the intervention. Such short booster sessions have a low participant burden, can be important reminders, and can easily be conducted in eHealth interventions. A review showed that combining web-delivered interventions with other delivery modes, such as SMS, telephone coaching, and emails, had stronger effects on behavior changes over time [21]. The process evaluation of this study [75] showed that 13% of the invited participants did not enter the Food4toddlers website at all, indicating some challenges in engaging all participants. Other deliveries might have been valuable toward achieving better engagement. However, personal contact is cost- and time-consuming, which limits distribution to the population at large [71]. Digital tailoring based on information about diet and physical activity provided by parents on the website or app, as done in the MINISTOP study [76], may contribute to better adherence. Even though the effect did not last after follow-up 1 in this study, there is still a possible public health benefit of increasing vegetable intake among children, even in small measures.

### Strengths and Limitations

Few parent-focused eHealth studies are exclusively web based and few target young children [13]. The participants in the Food4toddlers study represented all 19 Norwegian counties, which was possible because we used Facebook as the recruitment platform and had no face-to-face components in the intervention. The possibility of reaching a large and widespread population is one of the main benefits of using eHealth approaches [21]; however, we aimed for a larger sample in this study. Separate analyses for fruits and vegetables could also be viewed as a strength due to different consumption patterns and tastes [63-65] and are recommended for studies targeting young children [77,78]. A recently published review paper addressed the need to examine both variety and intake (ie, quantity) of fruits and vegetables due to the different findings regarding health outcomes; this review also revealed that such research was particularly lacking in young age groups [35].

A limitation of the study is the low generalizability of the findings due to the participants’ education level, which was higher than national figures [79]. It is conceivable that a more representative sample might have resulted in a larger intervention effect, as indicated in other studies [80,81]. Even though both parents were invited to participate, 287 of 291 (98.6%) participants were mothers. We do not know if our findings would have been different if more fathers were included. We aimed at recruiting a larger sample, but time and cost (ie, expensive Facebook advertisement) limited that. Therefore, we ended up with a more restricted sample size and, hence, lower statistical power than was planned for. It turned out to be challenging to recruit parents through Facebook when the children were around 10 months of age, possibly because parents in Norway often start working after their maternity leave around that time. Quantifying the dietary intake in grams and nutrient calculation might have added value to the assessments; however, portion size estimations were not recorded. Self-reported FFQs have limitations, especially in this age group where dietary habits are rapidly changing and the answers are solely dependent on the parents’ observations and suggestions [82]. A potential bias in intervention group reporting could be answering according to the perceived intention of the intervention (eg, higher intake of vegetables) [83]. The three questionnaires were delivered in different Nordic seasons—two in autumn-winter and one in winter-spring—which could have influenced the results, especially for fruit and vegetable intake. If so, the effect of the intervention would tend to be overrated. The digital approach limited the possibility of collecting objective measurements, leaving self-reported measures as the only option, which have limitations [84].

### Conclusions

In this study, we investigated the effects of the Norwegian Food4toddlers RCT. An intervention effect on the frequency of intake of vegetables was observed immediately after the 6-month intervention period ended. The difference was attenuated and no longer significant at the follow-up 2 time point, 6 months postintervention. The consumption of discretionary food increased over time in both groups.

Despite the potential of reaching a large population with limited resources, few eHealth interventions seeking to enhance children’s diets have targeted parents of toddlers at this key time in children’s food preference development. Our results show that there is a potential to improve aspects of young children’s diets utilizing this kind of intervention. To obtain long-term effects in eHealth interventions, longer durations should be considered along with tailoring in a digital or a personal form. Delivering short reminders after the end of the main content of the intervention may contribute to better adherence and is feasible in eHealth interventions.

## Appendix

Multimedia Appendix 1 CONSORT-EHEALTH V1.6.1 checklist for the Food4toddlers study.

## Abbreviations

CONSORT-EHEALTH: Consolidated Standards of Reporting Trials of Electronic and Mobile HEalth Applications and onLine TeleHealth
EMM: estimated marginal mean
FFQ: food frequency questionnaire
GEE: generalized estimating equation
ISRCTN: International Standard Randomised Controlled Trial Number
mHealth: mobile health
MINISTOP: Mobile-based intervention intended to stop obesity in preschoolers
RCT: randomized controlled trial
WHO: World Health Organization

## References

[1] Johnson BJ, Hendrie GA, Golley RK (2015). Reducing discretionary food and beverage intake in early childhood: A systematic review within an ecological framework. Public Health Nutr.

[2] McCarthy R, Kehoe L, Flynn A, Walton J (2020). The role of fruit and vegetables in the diets of children in Europe: Current state of knowledge on dietary recommendations, intakes and contribution to energy and nutrient intakes. Proceedings of the 13th European Nutrition Conference.

[3] Hodder R, O'Brien K, Tzelepis F, Wyse R, Wolfenden L (2020). Interventions for increasing fruit and vegetable consumption in children aged five years and under. Cochrane Database Syst Rev.

[4] WHO technical staff (2014). Increasing fruit and vegetable consumption to reduce the risk of noncommunicable diseases. World Health Organization.

[5] (2016). Report of the Commission on Ending Childhood Obesity.

[6] Aune D, Giovannucci E, Boffetta P, Fadnes L, Keum N, Norat T (2017). Fruit and vegetable intake and the risk of cardiovascular disease, total cancer and all-cause mortality-A systematic review and dose-response meta-analysis of prospective studies. Int J Epidemiol.

[7] Scaglioni S, De Cosmi V, Ciappolino V, Parazzini F, Brambilla P, Agostoni C (2018). Factors influencing children’s eating behaviours. Nutrients.

[8] Grimm KA, Kim SA, Yaroch AL, Scanlon KS (2014). Fruit and vegetable intake during infancy and early childhood. Pediatrics.

[9] Rose CM, Birch LL, Savage JS (2017). Dietary patterns in infancy are associated with child diet and weight outcomes at 6 years. Int J Obes.

[10] Bjelland M, Brantsæter AL, Haugen M, Meltzer HM, Nystad W, Andersen LF (2013). Changes and tracking of fruit, vegetables and sugar-sweetened beverages intake from 18 months to 7 years in the Norwegian mother and child cohort study. BMC Public Health.

[11] Larsen JK, Hermans RC, Sleddens EF, Engels RC, Fisher JO, Kremers SP (2015). How parental dietary behavior and food parenting practices affect children's dietary behavior. Interacting sources of influence?. Appetite.

[12] Faith MS, Van Horn L, Appel LJ, Burke LE, Carson JAS, Franch HA, Jakicic JM, Kral TV, Odoms-Young A, Wansink B, Wylie-Rosett J (2012). Evaluating parents and adult caregivers as “agents of change” for treating obese children: Evidence for parent behavior change strategies and research gaps. Circulation.

[13] Hammersley ML, Jones RA, Okely AD (2016). Parent-focused childhood and adolescent overweight and obesity eHealth interventions: A systematic review and meta-analysis. J Med Internet Res.

[14] Redsell SA, Edmonds B, Swift JA, Siriwardena AN, Weng S, Nathan D, Glazebrook C (2015). Systematic review of randomised controlled trials of interventions that aim to reduce the risk, either directly or indirectly, of overweight and obesity in infancy and early childhood. Matern Child Nutr.

[15] Sayakhot P, Carolan-Olah M (2016). Internet use by pregnant women seeking pregnancy-related information: A systematic review. BMC Pregnancy Childbirth.

[16] Slomian J, Bruyère O, Reginster J, Emonts P (2017). The internet as a source of information used by women after childbirth to meet their need for information: A web-based survey. Midwifery.

[17] Helle C, Hillesund ER, Wills AK, Øverby NC (2019). Examining the effects of an eHealth intervention from infant age 6 to 12 months on child eating behaviors and maternal feeding practices one year after cessation: The Norwegian randomized controlled trial Early Food for Future Health. PLoS ONE.

[18] Moon RY, Mathews A, Oden R, Carlin R (2019). Mothers' perceptions of the internet and social media as sources of parenting and health information: Qualitative study. J Med Internet Res.

[19] Litterbach E, Russell CG, Taki S, Denney-Wilson E, Campbell KJ, Laws RA (2017). Factors influencing engagement and behavioral determinants of infant feeding in an mHealth program: Qualitative evaluation of the growing healthy program. JMIR Mhealth Uhealth.

[20] Russell CG, Denney-Wilson E, Laws RA, Abbott G, Zheng M, Lymer SJ, Taki S, Litterbach EV, Ong K, Campbell KJ (2018). Impact of the growing healthy mHealth program on maternal feeding practices, infant food preferences, and satiety responsiveness: Quasi-experimental study. JMIR Mhealth Uhealth.

[21] Vandelanotte C, Müller AM, Short CE, Hingle M, Nathan N, Williams SL, Lopez ML, Parekh S, Maher CA (2016). Past, present, and future of eHealth and mHealth research to improve physical activity and dietary behaviors. J Nutr Educ Behav.

[22] Birch LL, Ventura AK (2009). Preventing childhood obesity: What works?. Int J Obes.

[23] Pearson N, Biddle SJ, Gorely T (2009). Family correlates of fruit and vegetable consumption in children and adolescents: A systematic review. Public Health Nutr.

[24] Lacy KE, Spence AC, McNaughton SA, Crawford DA, Wyse RJ, Wolfenden L, Campbell KJ (2019). Home environment predictors of vegetable and fruit intakes among Australian children aged 18 months. Appetite.

[25] Røed M, Hillesund ER, Vik FN, Van Lippevelde W, Øverby NC (2019). The Food4toddlers study - Study protocol for a web-based intervention to promote healthy diets for toddlers: A randomized controlled trial. BMC Public Health.

[26] (2021). Mat til minsten (Food4toddlers). University of Agder.

[27] (2019). SurveyXact. Rambøll.

[28] Brug J, Oenema A, Ferreira I (2005). Theory, evidence and intervention mapping to improve behavior nutrition and physical activity interventions. Int J Behav Nutr Phys Act.

[29] Kelder S, Hoelscher D, Perry C, Glanz K, Rimer BK, Viswanath K (2015). How individuals, environments, and health behaviors interact. Health Behavior: Theory, Research, and Practice. 5th edition.

[30] Petty R, Barden J, Wheeler S, DiClemente RJ, Crosby RA, Kegler MC (2009). The elaboration likelihood model of persuasion: Developing health promotions for sustained behavioral change. Emerging Theories in Health Promotion Practice and Research. 2nd edition.

[31] Mak TN, Prynne CJ, Cole D, Fitt E, Bates B, Stephen AM (2013). Patterns of sociodemographic and food practice characteristics in relation to fruit and vegetable consumption in children: Results from the UK National Diet and Nutrition Survey Rolling Programme (2008–2010). Public Health Nutr.

[32] Jacobs JD, Steffen L (2003). Nutrients, foods, and dietary patterns as exposures in research: A framework for food synergy. Am J Clin Nutr.

[33] Cena H, Calder PC (2020). Defining a healthy diet: Evidence for the role of contemporary dietary patterns in health and disease. Nutrients.

[34] Wirt A, Collins CE (2009). Diet quality – What is it and does it matter?. Public Health Nutr.

[35] Marshall AN, van den Berg A, Ranjit N, Hoelscher DM (2020). A scoping review of the operationalization of fruit and vegetable variety. Nutrients.

[36] Ramsay SA, Shriver LH, Taylor CA (2017). Variety of fruit and vegetables is related to preschoolers' overall diet quality. Prev Med Rep.

[37] Røed M, Vik FN, Hillesund ER, Lippevelde WV, Øverby NC (2020). Associations between parental food choice motives, health-promoting feeding practices, and infants' fruit and vegetable intakes: The Food4toddlers study. Food Nutr Res.

[38] (2018). Norwegian Mother, Father and Child Cohort Study (MoBa). Norwegian Institute of Public Health.

[39] Øverby N, Kristiansen A, Andersen L, Lande B (2009). Spedkost 12 Months: National Dietary Survey Among 12-Month-Old Children.

[40] Andersen L, Lande B, Trygg K, Hay G (2007). Validation of a semi-quantitative food-frequency questionnaire used among 2-year-old Norwegian children. Public Health Nutr.

[41] Jin F (2016). Questionnaire 5 When the Child Was 18 Months Old. The Norwegian Mother and Child Cohort Study (MoBa): Mother Questionnaire. Version 1.0.

[42] (2019). Survey of consumer expenditure. Statistics Norway.

[43] Campbell KJ, Crawford DA, Ball K (2006). Family food environment and dietary behaviors likely to promote fatness in 5-6-year-old children. Int J Obes.

[44] Kiefner-Burmeister AE, Hoffmann DA, Meers MR, Koball AM, Musher-Eizenman DR (2014). Food consumption by young children: A function of parental feeding goals and practices. Appetite.

[45] Harrison K, Liechty JM (2012). US preschoolers' media exposure and dietary habits: The primacy of television and the limits of parental mediation. J Child Media.

[46] Lanfer A, Hebestreit A, Ahrens W, Krogh V, Sieri S, Lissner L, Eiben G, Siani A, Huybrechts I, Loit H, Papoutsou S, Kovács E, Pala V, IDEFICS Consortium (2011). Reproducibility of food consumption frequencies derived from the Children's Eating Habits Questionnaire used in the IDEFICS study. Int J Obes (Lond).

[47] Cutler DM, Lleras-Muney A (2006). Education and Health: Evaluating Theories and Evidence.

[48] Angelopoulos P, Kourlaba G, Kondaki K, Fragiadakis GA, Manios Y (2009). Assessing children's diet quality in Crete based on Healthy Eating Index: The Children Study. Eur J Clin Nutr.

[49] Salazar A, Ojeda B, Dueñas M, Fernández F, Failde I (2016). Simple generalized estimating equations (GEEs) and weighted generalized estimating equations (WGEEs) in longitudinal studies with dropouts: Guidelines and implementation in R. Stat Med.

[50] Rasmussen M, Krølner R, Klepp KI, Lytle L, Brug J, Bere E, Due P (2006). Determinants of fruit and vegetable consumption among children and adolescents: A review of the literature. Part I: Quantitative studies. Int J Behav Nutr Phys Act.

[51] Bell ML, Fiero M, Horton NJ, Hsu C (2014). Handling missing data in RCTs: A review of the top medical journals. BMC Med Res Methodol.

[52] University of Agder (2020). UiA Open Research Data. University of Agder.

[53] (2019). Population. Statistics Norway.

[54] Christensen H, Griffiths KM, Farrer L (2009). Adherence in internet interventions for anxiety and depression. J Med Internet Res.

[55] Carstairs SA, Craig LC, Marais D, Bora OE, Kiezebrink K (2016). A comparison of preprepared commercial infant feeding meals with home-cooked recipes. Arch Dis Child.

[56] Kristiansen A, Andersen L, Lande B (2009). Småbarnskost 2 år: National Dietary Survey Among 2-Year-Old Children.

[57] Dovey TM, Staples PA, Gibson EL, Halford JC (2008). Food neophobia and ‘picky/fussy’ eating in children: A review. Appetite.

[58] Delisle Nyström C, Sandin S, Henriksson P, Henriksson H, Maddison R, Löf M (2018). A 12-month follow-up of a mobile-based (mHealth) obesity prevention intervention in pre-school children: The MINISTOP randomized controlled trial. BMC Public Health.

[59] Helle C, Hillesund ER, Wills AK, Øverby NC (2019). Evaluation of an eHealth intervention aiming to promote healthy food habits from infancy-The Norwegian randomized controlled trial Early Food for Future Health. Int J Behav Nutr Phys Act.

[60] Knowlden AP, Conrad E (2017). Two-year outcomes of the Enabling Mothers to Prevent Pediatric Obesity Through Web-Based Education and Reciprocal Determinism (EMPOWER) randomized control trial. Health Educ Behav.

[61] Chen J, Weiss S, Heyman MB, Cooper B, Lustig RH (2011). The efficacy of the web-based childhood obesity prevention program in Chinese American adolescents (Web ABC study). J Adolesc Health.

[62] Appleton KM, Hemingway A, Saulais L, Dinnella C, Monteleone E, Depezay L, Morizet D, Armando Perez-Cueto FJ, Bevan A, Hartwell H (2016). Increasing vegetable intakes: Rationale and systematic review of published interventions. Eur J Nutr.

[63] Glasson C, Chapman K, James E (2010). Fruit and vegetables should be targeted separately in health promotion programmes: Differences in consumption levels, barriers, knowledge and stages of readiness for change. Public Health Nutr.

[64] Appleton K, Hemingway A, Rajska J, Hartwell H (2018). Repeated exposure and conditioning strategies for increasing vegetable liking and intake: Systematic review and meta-analyses of the published literature. Am J Clin Nutr.

[65] Beauchamp GK, Mennella JA (2009). Early flavor learning and its impact on later feeding behavior. J Pediatr Gastroenterol Nutr.

[66] Hammersley ML, Okely AD, Batterham MJ, Jones RA (2019). An internet-based childhood obesity prevention program (Time2bHealthy) for parents of preschool-aged children: Randomized controlled trial. J Med Internet Res.

[67] Nyström CD, Sandin S, Henriksson P, Henriksson H, Trolle-Lagerros Y, Larsson C, Maddison R, Ortega FB, Pomeroy J, Ruiz JR, Silfvernagel K, Timpka T, Löf M (2017). Mobile-based intervention intended to stop obesity in preschool-aged children: The MINISTOP randomized controlled trial. Am J Clin Nutr.

[68] Vercammen KA, Frelier JM, Lowery CM, McGlone ME, Ebbeling CB, Bleich SN (2018). A systematic review of strategies to reduce sugar-sweetened beverage consumption among 0-year to 5-year olds. Obes Rev.

[69] van Grieken A, Vlasblom E, Wang L, Beltman M, Boere-Boonekamp MM, L'Hoir MP, Raat H (2017). Personalized web-based advice in combination with well-child visits to prevent overweight in young children: Cluster randomized controlled trial. J Med Internet Res.

[70] Webb KL, Lahti-Koski M, Rutishauser I, Hector DJ, Knezevic N, Gill T, Peat JK, Leeder SR (2006). Consumption of ‘extra’ foods (energy-dense, nutrient-poor) among children aged 16–24 months from Western Sydney, Australia. Public Health Nutr.

[71] Kohl LF, Crutzen R, de Vries NK (2013). Online prevention aimed at lifestyle behaviors: A systematic review of reviews. J Med Internet Res.

[72] Yavuz HM, van Ijzendoorn MH, Mesman J, van der Veek S (2014). Interventions aimed at reducing obesity in early childhood: A meta-analysis of programs that involve parents. J Child Psychol Psychiatr.

[73] Helle C, Hillesund ER, Omholt ML, Øverby NC (2017). Early food for future health: A randomized controlled trial evaluating the effect of an eHealth intervention aiming to promote healthy food habits from early childhood. BMC Public Health.

[74] Verjans-Janssen SRB, van de Kolk I, Van Kann DHH, Kremers SPJ, Gerards SMPL (2018). Effectiveness of school-based physical activity and nutrition interventions with direct parental involvement on children’s BMI and energy balance-related behaviors – A systematic review. PLoS ONE.

[75] Røed M, Vik F, Hillesund E, Van Lippevelde W, Medin A, Øverby NC (2020). Process evaluation of an eHealth intervention (Food4toddlers) to improve toddlers' diet: Randomized controlled trial. JMIR Hum Factors.

[76] Delisle C, Sandin S, Forsum E, Henriksson H, Trolle-Lagerros Y, Larsson C, Maddison R, Ortega FB, Ruiz JR, Silfvernagel K, Timpka T, Löf M (2015). A web- and mobile phone-based intervention to prevent obesity in 4-year-olds (MINISTOP): A population-based randomized controlled trial. BMC Public Health.

[77] Krølner R, Rasmussen M, Brug J, Klepp K, Wind M, Due P (2011). Determinants of fruit and vegetable consumption among children and adolescents: A review of the literature. Part II: Qualitative studies. Int J Behav Nutr Phys Act.

[78] Ramsay SA, Eskelsen AK, Branen LJ, Armstrong Shultz J, Plumb J (2014). Nutrient intake and consumption of fruit and vegetables in young children. Child Obes Nutr.

[79] (2018). Educational attainment of the population. Statistics Norway.

[80] Pinket A, De Craemer M, Huybrechts I, De Bourdeaudhuij I, Deforche B, Cardon G, Androutsos O, Koletzko B, Moreno L, Socha P, Iotova V, Manios Y, Van Lippevelde W (2016). Diet quality in European pre-schoolers: Evaluation based on diet quality indices and association with gender, socio-economic status and overweight, the ToyBox-study. Public Health Nutr.

[81] Spence AC, Campbell KJ, Lioret S, McNaughton SA (2018). Early childhood vegetable, fruit, and discretionary food intakes do not meet dietary guidelines, but do show socioeconomic differences and tracking over time. J Acad Nutr Diet.

[82] Lovell A, Bulloch R, Wall CR, Grant CC (2017). Quality of food-frequency questionnaire validation studies in the dietary assessment of children aged 12 to 36 months: A systematic literature review. J Nutr Sci.

[83] Delgado-Rodríguez M, Llorca J (2004). Bias. J Epidemiol Community Health.

[84] Schoeller DA (1995). Limitations in the assessment of dietary energy intake by self-report. Metabolism.

